# Assessing the Hepatic Safety of Epigallocatechin Gallate (EGCG) in Reproductive-Aged Women

**DOI:** 10.3390/nu15020320

**Published:** 2023-01-09

**Authors:** Hiba Siblini, Ayman Al-Hendy, James Segars, Frank González, Hugh S. Taylor, Bhuchitra Singh, Ainna Flaminia, Valerie A. Flores, Gregory M. Christman, Hao Huang, Jeremy J. Johnson, Heping Zhang

**Affiliations:** 1 Department of Obstetrics and Gynecology, University of Chicago, Chicago, IL 60637, USA; 2 Department of Gynecology and Obstetrics, Johns Hopkins University School of Medicine, Baltimore, MD 21205, USA; 3 Department of Obstetrics and Gynecology, University of Illinois Chicago, Chicago, IL 60607, USA; 4 Department of Obstetrics, Gynecology, and Reproductive Sciences, Yale University, New Haven, CT 06520, USA; 5 Department of Obstetrics and Gynecology, University of Florida College of Medicine, Gainesville, FL 32610, USA; 6 Department of Biostatistics, Yale University School of Public Health, New Haven, CT 06510, USA; 7 Department of Pharmacy Practice, University of Illinois Chicago, Chicago, IL 60607, USA

**Keywords:** fibroids, leiomyomas, green tea, epigallocatechin gallate, EGCG, safety study, infertility, dietary factors, nutrition, women health, gynecologic diseases

## Abstract

A similar abstract of the interim analysis was previously published in Fertility and Sterility. EPIGALLOCATECHIN GALLATE (EGCG) FOR TREATMENT OF UNEXPLAINED INFERTILITY ASSOCIATED WITH UTERINE FIBROIDS (PRE-FRIEND TRIAL): EARLY SAFETY ASSESSMENT. Uterine fibroids are the most common cause of unexplained infertility in reproductive-aged women. Epigallocatechin gallate (EGCG), a green tea catechin, has demonstrated its ability to shrink uterine fibroids in prior preclinical and clinical studies. Hence, we developed an NICHD Confirm-funded trial to evaluate the use of EGCG for treating women with fibroids and unexplained infertility (FRIEND trial). Prior to embarking on that trial, we here conducted the pre-FRIEND study (NCT 04177693) to evaluate the safety of EGCG in premenopausal women. Specifically, our aim was to assess any adverse effects of EGCG alone or in combination with an ovarian stimulator on serum liver function tests (LFTs) and folate level. In this randomized, open-label prospective cohort, participants were recruited from the FRIEND-collaborative clinical sites: Johns Hopkins University, University of Chicago, University of Illinois at Chicago, and Yale University. Thirty-nine women, ages ≥18 to ≤40 years, with/without uterine fibroids, were enrolled and randomized to one of three treatment arms: 800 mg of EGCG daily alone, 800 mg of EGCG daily with clomiphene citrate 100 mg for 5 days, or 800 mg of EGCG daily with Letrozole 5 mg for 5 days. No subject demonstrated signs of drug induced liver injury and no subject showed serum folate level outside the normal range. Hence, our data suggests that a daily dose of 800 mg of EGCG alone or in combination with clomiphene citrate or letrozole (for 5 days) is well-tolerated and is not associated with liver toxicity or folate deficiency in reproductive-aged women.

## 1. Introduction

Uterine fibroids (UFs) are the most common pelvic gynecological tumor, affecting more than 70% of reproductive-aged women [1]. UFs are the leading indication for hysterectomies performed in the United States and account for 39% of the total hysterectomies [2], with an estimated cost of up to USD 34.4 billion annually in the USA alone [3]. At present, a woman with symptomatic fibroids who seeks to get pregnant has no non-surgical, safe method of treatment that will concurrently manage her fibroid-related symptoms without impacting her chances of achieving a healthy, safe pregnancy. Furthermore, if myomectomy is performed, the rate of fibroid recurrence is high, up to 38% at 3 years postoperative [4] and may be followed by additional complications [5]. Uterine fibroids are a major health disparity issue, with an incidence that is nearly 3–4 times higher in African American women and are approximately twice as prevalent in Hispanic women compared to Caucasian women [6,7,8]. This debilitating disorder applies a disproportionate burden on women of color, and the associated challenges, such as infertility, might adversely affect their quality of life and well-being [9]. Thus, there is a crucial need to develop a new, safe, durable, and effective non-invasive treatment option for uterine fibroid-associated infertility.

### 1.1. Impact of Uterine Fibroids on Female Fertility

Uterine fibroids affect 30–77% of reproductive-aged women [10]. The actual prevalence may however be much higher, as many women with fibroids have few to no recognizable symptoms [11]. The live birth rate among women with fibroids is influenced by fibroid location: submucosal fibroids are associated with a 70% reduction in delivery rate (compared to women without fibroids); however, intramural fibroids reduce the delivery rate by approximately 30% [12]. Bulletti et al. supported the concept that fibroids impact fertility and evaluated spontaneous conception in infertile women with or without fibroids, yielding a significant difference in the pregnancy rates between the two groups (11% with fibroids versus 25% without fibroids) with up to three in vitro fertilization (IVF) cycles [13]. Nevertheless, with the lack of well-designed controlled clinical studies, evidence on the impact of fibroids on infertility is still complex, even with the abundance of observational studies. Donnez J and Jadoul P conducted a review of 106 articles of myomas and myomectomy and their impact on fertility and pregnancy outcomes in infertile women. The authors reported that regardless of these tumors’ characteristics, uterine fibroids were found to be the only abnormality in approximately 2.4% of infertile women [14].

### 1.2. Currently Available Treatment Options for Uterine Fibroids in Women Desiring Future Fertility

There are few therapies approved by the US Food and Drug Administration (FDA) for the treatment of fibroids: gonadotropin-releasing hormone (GNRH) agonists, recently approved GNRH antagonists, embolic agents for uterine artery embolization, magnetic resonance imaging-guided high-energy focused ultrasound ablation, and surgery [15]. Current management strategies for patients who desire future fertility are mainly centered around myomectomy, via the abdominal, vaginal, laparoscopic, or hysteroscopic approach [16]. Therapeutic drugs may offer a substitute for many symptomatic patients with fibroids who desire a more conservative management approach, with examples of such drugs including NSAIDs, combined oral contraceptives (OCPs), progestins, and levonorgestrel intrauterine system devices (IUDs), and approved (GnRH agonists such as Lupron and antagonists including Elagolix, Relugolix, and Linzagolix) or investigational agents (e.g., selective progesterone receptor modulators (SPRMs)); Mifepristone, Asoprisnil, Telapristone acetate, Ulipristal, and Vilaprisa; somatostatin analogues: Lanreotide. However, none of these compounds are fertility-friendly [17]. Myomectomy is often the recommended treatment option for symptomatic uterine fibroids in women who desire to preserve their fertility or in infertile women with uterine fibroids [18]. Nevertheless, surgery carries the risks of subsequent uterine rupture during labor [19], postoperative peritoneal adhesions, abnormal placentation [20], pre-term delivery [21], and increased caesarian delivery rates [22]. Therefore, there is a need for innovative non-surgical fertility-friendly treatment options for women suffering from uterine fibroid-associated infertility.

### 1.3. EGCG (Green Tea Extract) and Uterine Fibroids

Green tea leaves contain polyphenols, such as catechins and flavin-3-ols, examples of which include epicatechin, epicatechin gallate, epigallocatechin gallate (EGCG), and alkaloids. Catechins are the major components of tea phenols and represent approximately 30–42% of the dry weight of green tea [23]. The medical literature suggests that EGCG catechin is the most abundant and active compound responsible for most of green tea’s favorable research results. One study conducted by the United States Department of Agriculture reported that green tea has potent anti-neoplastic effects against a range of human tumor cells [24], with EGCG polyphenol demonstrating inhibitory abilities in key pathways of tumor growth. EGCG appears to block each stage of tumorigenesis by modulating the signaling pathways involved in cell proliferation, transformation, inflammation, apoptosis, oxidative stress, and invasion [24]. Studies by Al-Hendy et al. have reported that EGCG acts as an anti-uterine fibroid agent through the modulation of multiple signal transduction pathways through having a catechol-o-methyl-transferase (COMT) inhibitor effect, upregulating the expression of the bone morphogenic protein 2 (BMP2) gene, and decreasing TGF-β. As TGFβ3 is the main cytokine responsible for endometrial BMP-resistance in fibroid cells, this effect of EGCG would be expected to lead to improved endometrial receptivity [25,26,27]. Additionally, published work by Al-Hendy has demonstrated the utility of EGCG for inhibiting fibroid tumor formation in vivo in a nude mouse model [28].

### 1.4. Previous EGCG Clinical Trial in Women with Symptomatic Uterine Fibroids

To translate the positive EGCG-related anti-fibroid preclinical findings in human fibroid cell lines and in fibroid animal models, the efficacy and safety of EGCG on uterine fibroid burden and quality of life in women with symptomatic uterine fibroids were previously evaluated in an NIH-funded double-blinded, placebo-controlled randomized clinical trial (NCT 01311869) [29]. In this study, a total of 39 reproductive-aged women (between 18 and 50 years, day 3 serum follicle-stimulating hormone <12 IU/L) had symptomatic uterine fibroids. All subjects had at least one fibroid lesion that was 2 cm^3^ or larger in volume, as confirmed by transvaginal ultrasonography. The subjects were randomized to oral daily treatment with either 800 mg of decaffeinated EGCG or placebo (800 mg of brown rice) for 4 months, and uterine fibroid volumes were measured at the end of study by transvaginal ultrasonography. The study showed a significant reduction in (32.6%, *p* = 0.0001) the total uterine fibroid volume in subjects randomized to the green tea extract arm and a significant reduction in fibroid-specific symptom severity (32.4%, *p* = 0.0001) compared to the placebo group.

### 1.5. Green Tea Extracts and Hepatoxicity

The tea plant is used to make a variety of teas including black, oolong, and white tea. Native to southeast Asia, green tea is produced through rapidly steaming or pan-frying leaves to inhibit fermentation of monomeric catechins [30]. The steeping of green tea leaves releases a rich variety of water soluble catechins that include epicatechin-3-gallate (ECG), epigallocatechin (EGC), epigallocatechin-3-gallate (EGCG), and epicatechin (EC). Although consuming green tea as a beverage has a long history, the use of green tea extracts is a more recent development which has gained in popularity as an ingredient of dietary supplements. The USP (United States Pharmacopeia) identified 51 case reports associated with green tea extract and hepatotoxicity using daily doses ranging from 500 mg to 3000 mg daily (i.e., about 250 to 1800 mg of EGCG daily) [31]. The clinical evidence suggests that green tea extract taken on an empty stomach has an increased bioavailability and subsequently may increase the risk of liver injury [32].

### 1.6. Objective

Our primary objective is to assess the hepatic safety profile of EGCG in reproductive-aged women. In vitro and in vivo experimental studies, and case reports have demonstrated both hepatoprotective [33,34,35] and hepatotoxic [36,37,38] properties of green tea extract. A randomized, placebo-controlled study in healthy volunteers (eight subjects per group) in 2003 showed that EGCG administered at 800 mg/day for 4 weeks was safe and well-tolerated [34]. However, since research around a causal effect is equivocal, monitoring the effect of EGCG on liver enzymes was our primary aim in this pilot study. 

## 2. Materials and Methods

### 2.1. Study Design

**Figure 1:**
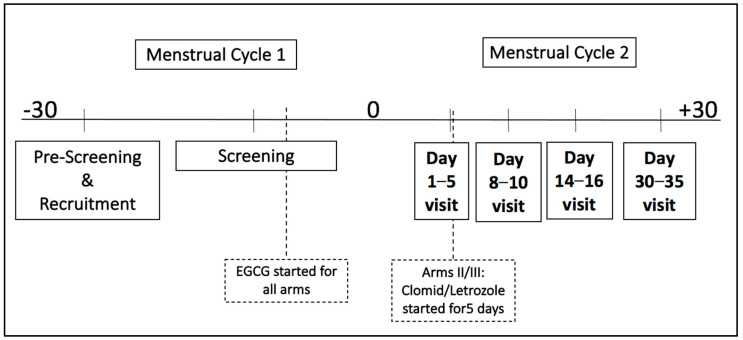
Study visit timeline. Treatment arm I is the green tea extract only for the duration of the study, arm II is the green tea extract and 5 days of clomiphene citrate, and arm III is the green tea extract and 5 days of letrozole. Day 1 is the start of the subject’s first menstrual cycle after the screening visit. EGCG: Epigallocatechin gallate.

This was a pharmacokinetic and safety study designed to assess the possible effects of a green tea extract (GTE) alone versus a GTE with a medication used for ovarian stimulation (clomiphene citrate or letrozole) given to women with or without fibroids under IND (#150951) from the US Food and Drug Administration. Green tea extract capsules were supplied by Beehive Botanicals Inc. (Hayward, Wisconsin), and each capsule contained on average, 400 mg of GTE with 45% EGCG. Subjects were instructed to take 4 capsules with food at breakfast daily, which is equivalent to a daily EGCG dose of 720 mg. GTE capsules were initiated after verifying screening eligibility and prior to the subject’s next cycle and were continued until the end of the study, which is around 30–35 days after the onset of their next menstrual cycle. An ovarian stimulation medication was started between cycle days 2–5 for subjects randomized to either regimen (clomiphene citrate or letrozole, treatment arms II and III, respectively), being 100 mg clomiphene citrate or 2.5 mg of letrozole for 5 days. A total of five visits were conducted throughout this study, including the screening visit followed by four study visits; liver function tests were monitored at every visit except at the mid-cycle ultrasound visit (Figure 1). Subjects were randomly assigned to one the three groups: EGCG alone, EGCG + clomiphene citrate, EGCG + Letrozole. Randomization was stratified by study site, age (18–29 and 30–40) and presence of fibroids. The protocol was reviewed and approved by a National Institutes of Health/National Institute of Child Health and Data Safety Monitoring Committee, with informed written consent provided by all the participants from each participating site according to an Institutional Review Board-approved protocol.

### 2.2. Inclusion/Exclusion Criteria

The goal of the inclusion and exclusion criteria was to identify non-pregnant or lactating women between the ages of 18 and 40 years old inclusively with or without fibroids. Subjects agreed to use barrier contraception throughout the duration of the study and were not users of hormonal medications, including oral contraceptives, nor of supplements with green tea extracts. Since this safety trial monitors liver function tests, subjects having any known liver disease, or a history of alcohol abuse were excluded from the study.

### 2.3. Study Conduct

After the provision of informed, written consent, study participants underwent a screening visit which included physical examination, measurements of their height, weight, and abdominal and hip circumference, and a gynecological exam, and a Papanicolaou smear as required per the current American Congress of Obstetricians and Gynecologists guidelines [39]. Blood was obtained to determine the inclusion/exclusion criteria, with samples being analyzed to conduct liver function and pregnancy tests and to determine estradiol and folate levels. A medical history survey was conducted at screening. After the screening visit, subjects called at the start of their next menstrual cycle (denoted as time 0 in Figure 1) and were then randomized to one of the three study arms. Liver function tests were monitored at every visit, except during the mid-cycle (day 14–16) ultrasound visit.

### 2.4. Data Management and Statistical Analysis

The Data Coordinating Center for this study was the Collaborative Center for Statistics in Science at Yale University in New Haven, Connecticut. The Data Coordinating Center was responsible for data management (including the data entered at each participating center) and data analysis. Since the number of patients for this study was minimal, descriptive statistics was used. Data are expressed as mean ± SD, or median (interquartile range) for the continuous variables within each treatment group, and the Kruskal–Wallis test was used for testing differences among the continuous variables among the three groups. The Chi-square test and Fisher’s exact test were used to test the differences in the categorical variables. Analysis was done using the SAS version 9.4 software. Significance was defined as a 2-sided *p* < 0.05.

## 3. Results and Analysis

### 3.1. Participant Disposition

**Figure 2:**
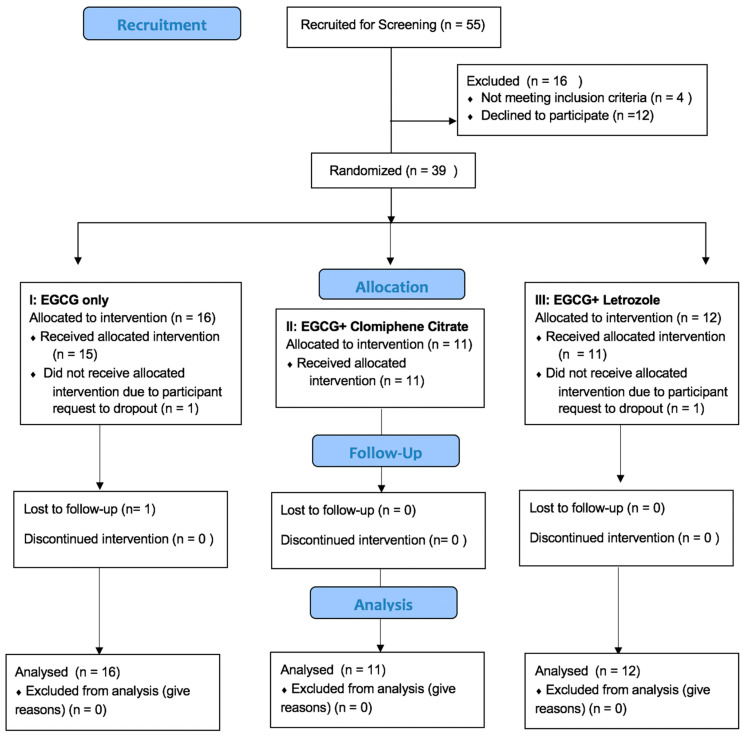
Flow chart of study population; EGCG: Epigallocatechin gallate.

Out of the 39 randomized participants, 36 completed all five of the visits required for the study. The reasons for drop-out included participant request (n = 2) and loss to follow up (n = 1). None attributed dropping out to adverse events. The flowchart of the study population is shown in Figure 2.

### 3.2. Demographics

Baseline demographics and participant information are presented in Table 1. On average, the women were 29 years old, primarily white (59.0%), and had a mean body mass index (BMI) of 27.2 kg/m^2^. In total, 15% of the participants had a previous diagnosis of uterine fibroids. No statistically significant differences in these characteristics were observed between participants randomized to the EGGC-only group compared to those randomized to the EGCG and clomid, or EGCG and letrozole groups. In addition, the mean baseline AST, ALT, bilirubin, and folate levels were all within the normal ranges (20.9 U/L, 16.9 U/L, 0.5 mg/dL, and 14.7 ng/mL, respectively), and were not statistically different between the three treatment groups.

### 3.3. Adverse Events

No participant experienced or reported a serious adverse event (AE) during the study. There has been a total of 12 AE reports related to abdominal discomfort and loose stools, nausea, and insomnia. The groups did not statistically differ in the reported AEs or in their severity. A detailed list of AE’s is presented in Table 2.

### 3.4. Liver Safety and Folate Levels at the End of the Study

Liver function tests and the determination of folate levels were performed throughout the study duration, which ranged between 4 to 6 weeks after the initiation of EGCG among the subjects stratified by treatment arm. Table 3 shows the mean values at the end of the study, and Table 4 shows the changes in the safety labs from screening until the last visit. All of the mean values of the liver function tests between the three groups remained in the normal reference range, and there were no significant differences observed among subjects in the three treatment arms. We adopted Hy’s law to identify any drug-induced liver injury [40,41], and have observed no cases of Hy’s Law among our subjects, as shown in Table 5.

The mean folate levels also remained within the reference range, and no subject demonstrated a deficiency in the folate level at the end of the study, with no subject being on any folic acid supplementation. Moreover, the mean mid-cycle endometrial thickness averaged between 8.6 in the EGCG-only group, and 11.1 in the EGCG + clomid group, with the changes observed from screening the endometrial thickness being non-significant in the three treatment groups.

## 4. Discussion

Green tea is one of the world’s most commonly consumed beverage [42]. In Japan, green tea extract beverages are marked as “Healthy green tea” or “Healthy water”, containing 540 mg of total catechins per daily serving [43]. A cross-sectional study of effects of green tea found that green tea was associated with many health benefits, such as the prevention of obesity, diabetes, and neurodegenerative diseases [44]. Moreover, the cancer chemo-preventive benefits of green tea have been shown in many target organs, including in the liver, lung, mammary glands, gastrointestinal tract, and skin [45,46,47]. The principal, most abundant, and most potent antioxidative active polyphenol in green tea is EGCG, which comprises approximately two thirds of total green tea catechins [48]. Another polyphenol tea catechin which is similar in structure to EGCG is epicatechin which has been shown to attenuate hepatic sinusoidal obstruction syndrome in rats by inhibiting liver oxidative injury [49].

In this study, we aimed to explore the safety profile of EGCG in healthy young women. Previously, some concerns had been raised in observational studies regarding a possible connection between green tea extract consumption and hepatotoxicity. Such studies suffered from several limitations, such as small participant numbers and supra-pharmacological doses of EGCG (greater than 800 mg/day), and were also performed in persons with Down’s syndrome, 56% males, post-menopausal women, patients with prostate cancer, and patients with type 2 diabetes [43]. Until recently, there were no data from a randomized clinical trial regarding safety of EGCG in healthy reproductive-aged women. Before launching a larger study to evaluate the utility of EGCG to enhance fertility in women suffering from unexplained infertility and uterine fibroids in which EGCG will be used in combination with ovarian stimulation, we evaluated the possible effects of EGCG given in combination with clomiphene or letrozole on liver function tests. In this study, standardized and low-caffeine green tea extract containing 720 mg of EGCG, an amount almost equivalent to 8 average 8 oz cups, was consumed once daily for at least a month with or without medications used for ovarian stimulation (clomiphene citrate or letrozole) taken for 5 days. The analysis demonstrated that of the 39 enrolled participants of which 36 subjects completed all study visits, none showed any signs of drug-induced liver injury according to Hy’s law. The three drop-out participants voluntarily decided to terminate their participation, which was not due to any adverse events. Based on the reported adverse effects and clinical laboratory safety data, the study treatment arms, and dosing schedule was proven to be safe and well-tolerated by the study participants for at least 1 month, and no significant changes were observed in the liver function tests throughout the study duration. Additionally, we do not expect EGCG to accumulate in the body when following a once-daily schedule on the basis of its short plasma half-life [50]. 

Our study analysis is in accordance with the results of a previous one month long randomized placebo-controlled clinical trial, in which 40 healthy male and female participants (≥18 years of age) received 800 mg of EGCG once daily or 400 mg of EGCG twice daily in the form of pure EGCG or Polyphenon E [35]. The authors concluded that these doses and this frequency were safe and well-tolerated.

In 2018, following a request from the European Commission to the European Food Safety Authority (EFSA), the Scientific Panel on Food Additives and Nutrient Sources added to Food (ANS) was asked to provide a scientific opinion on the safety of green tea catechins [43]. The panel concluded that there was no evidence of hepatotoxicity below 800 mg of EGCG/day for up to 12 months when taken with food according to the 38 intervention studies reviewed. They also reported that rare cases of liver injury have been observed after the consumption of green tea infusions, which where most likely linked to idiosyncratic reactions.

The United States Pharmacopeia has also updated its ongoing review on the safety of green tea extracts and has included a cautionary labeling requirement in its Powdered Decaffeinated Green Tea Extract monograph that reads “Do not take on an empty stomach. Take with food. Do not use if you have a liver problem and discontinue use and consult a healthcare practitioner if you develop symptoms of liver trouble, such as abdominal pain, dark urine, or jaundice (yellowing of the skin or eyes).” [31]. The USP has elucidated that the recognized factors that can contribute to hepatotoxic effects are the concentration of catechins in certain GTE-containing products, the bolus dose provided, the presence of elemental impurities including elemental metals, and the intake of GTE under fasting conditions. More specifically, it has been shown that under fasting conditions, the systemic plasma catechin concentration is significantly higher compared to when taken on a fed state [31], which was the condition under which EGCG was administered in this study. All subjects in this study were instructed to take the EGCG pills with food at breakfast. The results of our study are also congruent with those of the Minnesota Green Tea trial which concluded that the daily consumption of 843 mg of EGCG was generally well tolerated in a group of Caucasian postmenopausal women [51]. A very small subset of postmenopausal women had adverse effects related to liver enzymes however none showed clinical significance [51].

Moreover, measurement of the mid-cycle endometrium thickness revealed a range between 5.7 mm and 12 mm, and the mean was higher than 8.0 mm for all subgroups. This was reassuring since planned studies will use EGCG in intrauterine insemination (IUI) cycles with ovarian stimulation. Nevertheless, the effect of endometrial thickness during ovarian stimulation with IUI has been previously difficult to elucidate clearly. Still, the general consensus in the literature is that, although average women who conceive have a thicker endometrium, no significant association can be found [52]. A systematic review by Weiss et al. found no association between endometrial thickness and pregnancy rates following ovarian stimulation through intrauterine insemination in couples with unexplained or mild male infertility [53]. Furthermore, a prospective cohort analysis of the Reproductive Medicine Network’s Assessment of Multiple Intrauterine Gestations from Ovarian Stimulation (AMIGOS) randomized controlled trial represents the largest data cohort examining this question and concluded that endometrial thickness did not have any predictive values for endometrial receptivity and is not an independent a reliable determinant of live birth [54]. Additionally, differences in the mean endometrial thickness when taking clomiphene citrate and letrozole (8.9 vs. 9.5 mm) proved to be nonsignificant during subgroup analysis. 

The limitation of this study may be that it was not restricted to women with fibroids. Additionally, the study participants were in relatively good health and had no major comorbidities. Likewise, women with a previously known liver disease, binge drinkers, or women with a history of alcohol abuse, which is defined as more than 14 drinks per week, were excluded from the study to avoid potential confounding effects of liver disease and alcohol on liver enzymes. There may be other variables that could influence the observed effect of green tea extract on liver enzymes such as obesity [37]. No significant correlation between BMI and liver enzymes was seen in our study, or an elevation in liver enzymes, although this could be investigated further in a larger population set.

## 5. Conclusions

In the US, the fertility of reproductive-aged women with fibroids is a growing concern. A previous clinical study has shown a beneficial effect of EGCG on decreasing fibroid size and proliferation [25]. In this randomized phase I study, we have shown that 720 mg of EGCG taken by healthy reproductive-aged women daily with food for at least one month, alone or in combination with clomiphene citrate or letrozole for 5 days, is not associated with drug-induced liver injury. In our next phase 2 FRIEND study (NCT05364008), we will study the effects of EGCG on pregnancy outcomes in women with fibroids and unexplained infertility. 

## Figures and Tables

**Table 1:** Demographic characteristics of randomized patients.

	EGCG	EGCG + Clomiphene	EGCG + Letrozole	All	*p*-Value for Comparison among Three Groups
Age (years)	16 29.6 ± 6.3 28.5 (24.0 to 35.5)	11 27.2 ± 6.6 28.0 (19.0 to 34.0)	12 29.3 ± 6.3 27.0 (25.0 to 34.5)	39 28.8 ± 6.3 28.0 (24.0 to 34.0)	0.675
BMI (kg/m^2^)	16 26.1 ± 6.8 24.3 (22.1 to 29.3)	11 27.4 ± 5.7 28.3 (22.4 to 31.0)	12 28.4 ± 9.5 25.0 (22.6 to 30.9)	39 27.2 ± 7.3 25.0 (22.2 to 30.6)	0.709
Hip circumference (cm)	16 112.9 ± 35.1 104.0 (95.5 to 112.5)	10 103.6 ± 18.1 108.2 (90.0 to 116.0)	12 106.2 ± 19.4 105.3 (92.0 to 120.0)	38 108.4 ± 26.6 104.8 (94.0 to 116.0)	0.946
Waist circumference (cm)	16 90.7 ± 27.0 80.5 (75.5 to 99.0)	10 86.0 ± 13.4 89.8 (75.0 to 97.0)	12 88.6 ± 21.0 88.2 (71.6 to 97.3)	38 88.8 ± 21.8 84.1 (75.0 to 97.0)	0.991
Ethnicity					1.000
Hispanic or Latino	2/16 (12.5%)	2/11 (18.2%)	2/12 (16.7%)	6/39 (15.4%)	
Non-Hispanic	14/16 (87.5%)	9/11 (81.8%)	10/12 (83.3%)	33/39 (84.6%)	
Race					0.763
White	11/16 (68.8%)	6/11 (54.5%)	6/12 (50.0%)	23/39 (59.0%)	
Black	2/16 (12.5%)	2/11 (18.2%)	1/12 (8.3%)	5/39 (12.8%)	
Asian	2/16 (12.5%)	2/11 (18.2%)	4/12 (33.3%)	8/39 (20.5%)	
Native Hawaiian/Pacific Islander	1/16 (6.3%)	0/11 (0.0%)	0/12 (0.0%)	1/39 (2.6%)	
I prefer not to answer	0/16 (0.0%)	1/11 (9.1%)	1/12 (8.3%)	2/39 (5.1%)	
Have ever been diagnosed with fibroids	3/16 (18.8%)	2/11 (18.2%)	1/12 (8.3%)	6/39 (15.4%)	0.742
Have had endometriosis	1/15 (6.7%)	0/10 (0.0%)	0/11 (0.0%)	1/36 (2.8%)	1.000
History of smoking					0.949
Never	13/16 (81.3%)	9/10 (90.0%)	9/11 (81.8%)	31/37 (83.8%)	
Current	2/16 (12.5%)	0/10 (0.0%)	1/11 (9.1%)	3/37 (8.1%)	
Former	1/16 (6.3%)	1/10 (10.0%)	1/11 (9.1%)	3/37 (8.1%)	
History of alcohol use					0.642
Never	1/16 (6.3%)	1/11 (9.1%)	3/12 (25.0%)	5/39 (12.8%)	
Current	14/16 (87.5%)	9/11 (81.8%)	8/12 (66.7%)	31/39 (79.5%)	

EGCG: Epigallocatechin gallate; BMI: Body Mass Index.

**Table 2:** Adverse events reported by participants.

	EGCG	EGCG + Clomiphene	EGCG + Letrozole	All	*p*-Value for Comparison among Three Groups
≥1 Adverse events	2/16 (12.5%)	4/11 (36.4%)	6/12 (50.0%)	12/39 (30.8%)	0.083
Abdominal discomfort and loose stool	0/16 (0.0%)	0/11 (0.0%)	1/12 (8.3%)	1/39 (2.6%)	0.590
Diarrhea	1/16 (6.3%)	0/11 (0.0%)	0/12 (0.0%)	1/39 (2.6%)	1.000
Dizziness	0/16 (0.0%)	1/11 (9.1%)	0/12 (0.0%)	1/39 (2.6%)	0.282
Facial flushing, muscle ache, and diarrhea after letrozole	0/16 (0.0%)	0/11 (0.0%)	1/12 (8.3%)	1/39 (2.6%)	0.590
Greenish discharge	0/16 (0.0%)	1/11 (9.1%)	0/12 (0.0%)	1/39 (2.6%)	0.282
Headaches	1/16 (6.3%)	0/11 (0.0%)	0/12 (0.0%)	1/39 (2.6%)	1.000
Insomnia	0/16 (0.0%)	1/11 (9.1%)	1/12 (8.3%)	2/39 (5.1%)	0.503
Kidney pain	0/16 (0.0%)	0/11 (0.0%)	1/12 (8.3%)	1/39 (2.6%)	0.590
Loose stool	0/16 (0.0%)	0/11 (0.0%)	1/12 (8.3%)	1/39 (2.6%)	0.590
Low mood	0/16 (0.0%)	1/11 (9.1%)	0/12 (0.0%)	1/39 (2.6%)	0.282
Migraine after vaccine	1/16 (6.3%)	0/11 (0.0%)	0/12 (0.0%)	1/39 (2.6%)	1.000
Nausea with EGCG	0/16 (0.0%)	1/11 (9.1%)	1/12 (8.3%)	2/39 (5.1%)	0.503
Pelvic cramps, dull and intermittent aching after letrozole	0/16 (0.0%)	0/11 (0.0%)	1/12 (8.3%)	1/39 (2.6%)	0.590
Vomiting and diarrhea after clomid	0/16 (0.0%)	1/11 (9.1%)	0/12 (0.0%)	1/39 (2.6%)	0.282

EGCG: Epigallocatechin gallate.

**Table 3:** End-of-study lab results.

Arms	Label	Mean	Std Dev	Lower Quartile	Upper Quartile
EGCG n = 14	ALT (U/L)	18.8	13.5	12.0	23.0
	AST (U/L)	19.6	6.9	15.0	25.0
	Direct bilirubin (mg/dL)	0.2	0.1	0.1	0.2
	Total bilirubin (mg/dL)	0.5	0.6	0.2	0.4
	Mid-Cycle Endo thickness (mm)	8.6	4.4	5.4	11.2
	Folate (U/L)	12.8	4.3	8.5	15.5
EGCG + Clomiphene n = 11	ALT (U/L)	18.6	9.6	12.0	23.0
	AST (U/L)	28.3	25.5	16.0	28.0
	Direct bilirubin (mg/dL)	0.2	0.1	0.1	0.2
	Total bilirubin (mg/dL)	0.4	0.2	0.3	0.6
	Mid-Cycle Endo thickness (mm)	11.1	4.0	7.8	12.0
	Folate (U/L)	13.6	4.9	10.1	17.9
EGCG + Letrozole n = 11	ALT (U/L)	16.6	9.6	10.0	23.0
	AST (U/L)	18.8	4.6	15.0	23.0
	Direct bilirubin (mg/dL)	0.2	0.1	0.1	0.2
	Total bilirubin (mg/dL)	0.5	0.4	0.3	0.6
	Mid-Cycle Endo thickness (mm)	8.8	3.6	5.7	9.7
	Folate (U/L)	10.3	4.0	7.3	12.6

ALT: alanine transaminase; AST: aspartate aminotransferase; EGCG: Epigallocatechin gallate.

**Table 4:** Changes in safety labs from the screening to the last study visit.

	EGCG	EGCG + Clomiphene	EGCG + Letrozole	All	*p*-Value for Comparison among Three Groups
Change in ALT/SGPT (U/L)	n = 15 1.1 ± 5.1	11 4.4 ± 11.7	12 −1.0 ± 7.6	38 0.5 ± 8.4	0.237
Change in AST/SGOT (U/L)	15 −3.8 ± 11.0	11 7.5 ± 27.9	12 −0.5 ± 4.4	38 0.5 ± 16.9	0.348
Change in direct bilirubin (mg/dL)	15 0.0 ± 0.1	10 −0.1 ± 0.1	12 0.0 ± 0.0	37 −0.0 ± 0.1	0.124
Change in total bilirubin (mg/dL)	15 0.1 ± 0.2	11 −0.0 ± 0.0	12 0.0 ± 0.1	38 0.0 ± 0.2	0.508
Change in folate (ng/mL)	14 −1.6 ± 4.0	10 −3.7 ± 4.3 *	10 −1.3 ± 3.3	34 −2.1 ± 3.9	0.716
Change in endometrial thickness from screening to mid-cycle (mm)	13 −1.9 ± 4.3	11 1.4 ± 2.0	11 0.6 ± 2.7	35 −0.0 ± 3.6	0.146

EGCG: Epigallocatechin gallate. Values are shown as n (top), mean ± SD (bottom). * Statistically significant changes (*p* < 0.05) based on the signed-rank test.

**Table 5:** Safety summary of subjects with liver tests meeting predefined limits of change per Hy’s law *.

Liver Function Test	Screening (n = 39)	Visit 1 (n = 38)	Visit 2 (n = 36)	Visit 4 (n = 36)
ALT or AST ≥ 3×ULN	0	0	0	0
BILI ≥ 2×ULN	0	0	0	0

ALT: alanine transaminase; AST: aspartate aminotransferase; EGCG: Epigallocatechin gallate. BILI: Total bilirubin. * Based on the Assessment of Drug-Induced Liver Injury (DILI) per the FDA [40].

## Data Availability

Raw data were generated at the Data Consortium Center at Yale University. Derived data supporting the findings of this study are available from the corresponding author on request. The data are not publicly available due to containing information that could compromise the privacy of participants.
